# Intravenous anidulafungin followed optionally by oral voriconazole for the treatment of candidemia in Asian patients: results from an open-label Phase III trial

**DOI:** 10.1186/1471-2334-13-219

**Published:** 2013-05-15

**Authors:** Piroon Mootsikapun, Po-Ren Hsueh, Deepak Talwar, Vilma M Co, Viraj Rajadhyaksha, Moh-Lim Ong

**Affiliations:** 1 Department of Medicine, Srinagarind Hospital, Khon Kaen, Thailand; 2 Departments of Laboratory Medicine and Internal Medicine, National Taiwan University Hospital, National Taiwan University College of Medicine, Taipei, Taiwan; 3 Metro Hospitals, Noida, India; 4 Makati Medical Center, Makati City, Philippines; 5 Medical Research Division, Pfizer Ltd, Jogeshwari Mumbai, India; 6 Pfizer Inc., New York, NY, USA

**Keywords:** Anidulafungin, Candidemia, Asia

## Abstract

**Background:**

Candidemia is a significant cause of morbidity and mortality in hospitalized patients, particularly in Asia. Anidulafungin has been reported to be an effective treatment for candidemia in Western populations, but little is known about its efficacy in Asian patients, where the clinical presentation and epidemiology may be different.

**Methods:**

An open-label study of anidulafungin for the treatment of candidemia was recently conducted in several Asian countries. Treatment was initiated with intravenous anidulafungin, given for at least 5 days, with the option to complete treatment with oral voriconazole. The primary endpoint was global (clinical and microbiological) response, and the primary analysis was the proportion of patients in the modified intent-to-treat population with successful global response at the end of therapy. Secondary analyses included proportion with successful global response in clinically relevant patient subgroups. The safety and tolerability profile of anidulafungin and voriconazole in this population was also investigated.

**Results:**

Forty-three patients were studied, including 42 in the modified intent-to-treat population. Eighteen patients were > 65 years, the largest age subgroup, and 21 had central venous catheters. The most common *Candida* species causing infection were *C. tropicalis* (n = 18) and *C. albicans* (n = 10). In the primary analysis, 73.8% had a successful global response at end of therapy. Success rates in subgroups were: 72.2% for *C. tropicalis* and 71.4% for *C. albicans* infection, 58.8% for patients > 65 years, and 81.0% for patients with central venous catheters. Safety and tolerability were comparable with the known profiles for anidulafungin (and voriconazole).

**Conclusions:**

Although the epidemiology of *Candida* infections was different in this open-label study, the efficacy of anidulafungin in Asian patients with documented candidemia was consistent with previous studies in Western populations. No new safety concerns were identified.

**Trial registration:**

http://www.clinicaltrials.gov identifier NCT00537329

## Background

*Candida* species are the main cause of invasive fungal disease, affecting a wide variety of patient populations, including critically ill patients, patients with hematological malignancies, elderly hospitalized patients, solid-organ transplant recipients, and adult diabetic patients [1]. *C. albicans* remains the most common pathogen causing candidemia and invasive candidiasis (C/IC), both worldwide [1,2] and in Asia [3], but other species are becoming increasingly common [1,4-7]. Moreover, species distribution and susceptibility to antifungal agents, show considerable geographic variation [3]. Therefore, knowledge of local epidemiology is important for the effective management of candidemia.

Crude mortality rates associated with C/IC are substantial, ranging from 30% to 81% according to recent reports [2,4]. Mortality varies according to patient age, pre-existing comorbidities, and the causative *Candida* species; the highest mortality rates are observed with *C. krusei,* followed by *C. tropicalis* and *C. glabrata*[2,8].

Regional data for C/IC in Asia are scarce. A retrospective review of admissions to a Korean intensive care unit reported an incidence of candidemia of 9.1 cases per 1000 admissions, with a notably high crude mortality of 96%, due to a high proportion of patients with malignancy [9]. Four centers in Taiwan reported crude mortality ranging from 43.1% to 60.7% [10], and a tertiary care center in North India reported a decrease in mortality rate from 72.2% to 47% over a 5-year period as awareness of disease prevalence increased [11].

Fluconazole is the most commonly used candidemia therapy, but susceptibility to fluconazole varies, both by *Candida* species and by geographical region [3]. Thus, other antifungal agents are also required that are more effective than fluconazole against *Candida*, particularly in regional populations where *C. albicans* is no longer the most common species causing infection.

The echinocandin anidulafungin is an effective alternative to fluconazole, demonstrating superiority to fluconazole for the treatment of C/IC in a pivotal clinical trial by Reboli *et al.*[12]. However, clinical studies of anidulafungin have so far been conducted mostly in North America and Europe, with few Asian patients studied [12-14]. Racial groups may differ in terms of epidemiology, disease presentation, drug tolerability, and response to treatment [8,15-19]. Thus, assessment of potential clinically relevant differences between Asian and Western populations treated with anidulafungin is required. Moreover, the extended-spectrum triazole voriconazole has been reported to have greater *in vitro* activity against most *Candida* species compared with fluconazole [3].

This study aimed primarily to evaluate the efficacy and safety of intravenous (i.v.) anidulafungin for the treatment of documented candidemia in a broad and diverse population of Asian patients, with Acute Physiology and Chronic Health Evaluation (APACHE II) scores ≤ 20. The activity of anidulafungin was investigated across a range of pre-defined patient subgroups. In addition, the relationship between response to treatment and β-D glucan levels was investigated. Step-down therapy to oral voriconazole following at least 5 days of i.v. anidulafungin was permitted, to investigate whether treatment success rates comparable with fluconazole could be achieved [3], whilst providing flexible treatment options, such as reducing the length of i.v. therapy.

## Methods

### Study design

This was a prospective Phase III, 12-week, open-label, multicenter, non-comparative study (January 2008 to March 2009). The trial was performed in accordance with the Declaration of Helsinki and International Conference on Harmonisation Good Clinical Practice guidelines; each center was also approved by the local institutional review board (IRB) (please see Additional file 1 for full details of the IRBs). Periodic monitoring visits were performed by the study sponsor or approved agents. Full ethical approval was obtained at each participating center, and all patients provided written informed consent prior to screening. Patients could withdraw from the trial at any time. The trial was registered on http://www.clinicaltrials.gov under the identifier NCT00537329.

### Patients

Patients were eligible for participation if they had at least one blood culture positive for *Candida* species (within 96 h of starting study treatment), and clinical evidence of infection within 48 h prior to study enrollment. They were required to be at least 18 years of age, of Asian race, and to have an APACHE II score of ≤ 20. Patients were excluded in the event of: suspected *Candida* osteomyelitis, endocarditis, meningitis, endophthalmitis, septic thrombophlebitis, or hepatosplenic candidiasis; administration of > 48 h of systemic antifungal therapy within 2 weeks before enrollment; previous history of anidulafungin treatment; hypersensitivity to any echinocandins or azole therapy; an estimated life expectancy of < 72 h; and pregnancy.

### ITT population

The intent-to-treat (ITT) population comprised all patients who received at least one dose of anidulafungin, and was used for the safety analysis.

### MITT population

The modified ITT (MITT) population comprised all ITT patients with a confirmed diagnosis of candidemia and was used for the efficacy analysis.

### Per-protocol population

The per-protocol population comprised MITT patients who were compliant with the study protocol, i.e. patients who received at least 5 days of i.v. anidulafungin (unless deemed a treatment failure after three doses of study medication); had an overall study drug compliance (as measured by pill count) of ≥ 75% and ≤ 120% for oral voriconazole; and did not receive any concomitant systemic antifungal agents.

### Study treatment

Patients received 200 mg i.v. anidulafungin as a single loading dose on Day 1, followed by 100 mg i.v. anidulafungin once daily thereafter for a minimum of 5 days. After 5 days, patients were allowed to step down to 200 mg twice-daily (BID) oral voriconazole (or 100 mg BID if body weight was < 40 kg). Criteria for step-down therapy were as follows: negative blood culture for *Candida* species; resolution or significant improvement of clinical signs/symptoms of acute invasive fungal disease, and no clinical evidence of invasive fungal disease, as assessed by the investigator; absence of neutropenia (i.e. absolute neutrophil count ≤ 500/mm^3^); hemodynamically stable; patient was able to take oral medication and had a functional gastrointestinal tract; and did not have any contraindications (including contraindicated concomitant medications) to voriconazole. If these conditions were not fulfilled, the patient was continued on anidulafungin until alternative systemic antifungal therapy was required. Treatment with either anidulafungin only or anidulafungin followed by voriconazole was continued as deemed appropriate by the investigator, up to a maximum of 42 days.

Concomitant systemic antifungal agents were not permitted during the study, although topical antifungal agents were allowed at the investigators’ discretion. All other treatments (including antimicrobial and antiviral agents) indicated for the primary illness and supportive medications were continued throughout the study.

### Laboratory monitoring

Blood cultures were taken on Days 1–6, or until a negative culture for *Candida* species was obtained. If the blood culture remained positive on Day 6, a repeat blood culture was carried out on Day 7 and then weekly thereafter, until a negative result was reported. The presence of *Candida* species was initially determined at local laboratories. Blood samples for the Fungitell β-D glucan assay (Associates of Cape Cod, East Falmouth, MA, USA) were analyzed by a single reference laboratory for the study [20]. Batch samples, prepared by the local laboratory, were sent to a central laboratory in each participating country for confirmation of pathogen identification and susceptibility testing to anidulafungin and voriconazole. Isolates with an anidulafungin minimum inhibitory concentration (MIC) of ≤ 2 μg/ml were considered susceptible, while those with an MIC of > 2 μg/ml were considered non-susceptible based on Pfaller *et al.*[21]. Voriconazole breakpoints used were ≤ 1 μg/ml (susceptible), > 1 to 4 μg/ml (susceptible dose-dependent), and > 4 μg/ml (resistant) based on Pfaller *et al.*[22].

### Efficacy endpoints

The primary endpoint of the study was the proportion of patients in the MITT population with successful global response at end of all treatment (EOT) on or before Day 42. Global response was deemed successful if there was both clinical success (defined as cure – resolution of signs and symptoms of candidemia, or significant improvement – incomplete but significant resolution of signs and symptoms of candidemia) and microbiological success (defined as eradication if follow-up culture result is negative for *Candida* species, or presumed eradication if follow-up blood cultures were not available, but the clinical outcome was defined as success). Failure of response was defined as no significant improvement in signs and symptoms, death due to candidemia, or positive follow-up blood culture result. Indeterminate response included death not attributable to candidemia, or patient lost to follow-up. Missing values for patients who withdrew prematurely from the study with a documented failure were imputed using the last observation carried forward (LOCF) approach. To ensure consistency across studies of similar design conducted globally, a decision was made post hoc to include missing and indeterminate values as failures.

Secondary efficacy endpoints included: global response rate and clinical and microbiological responses at the end of i.v. anidulafungin treatment (EOIT), at 2 and 6 weeks follow-up visits after EOT and at the end of 12 weeks from baseline; time to death, mortality attributed to candidemia and all-cause mortality. Global response rates at EOT were assessed in pre-defined patient subgroups and in patients with risk factors for candidemia. Pre-defined subgroups included neutropenic status and *Candida* species isolated at baseline. Risk factors for candidemia included central venous catheter, renal insufficiency, post-surgery status, age ≥ 65 years, chemotherapy within the previous 3 months, and solid organ transplantation.

### Safety assessments

The safety and tolerability analyses of anidulafungin were conducted throughout the study on the ITT population. Safety assessments were measured using adverse-event (AE) monitoring, clinical laboratory parameters, fundoscopy, physical and neurological examination, and vital-sign measurements.

All AEs and serious AEs were reported to the investigator and noted in the case report form, using the Medical Dictionary for Regulatory Activities coding system (Version 12.0). The investigator was required to assess the causality and severity of the AE.

### Statistical analysis

It was planned to enroll 100 patients for this study, assuming an overall response rate of 75%, the 95% confidence interval (CI) for the percentage of patients responding to treatment would range from 66.3% to 83.7%, allowing for 5% non-evaluability based on the Reboli *et al.* study [12].

For the global, clinical, and microbiologic success rates in the MITT population, a two-sided exact (Clopper-Pearson) 95% CI was calculated [23]. In addition, descriptive summaries were provided for all secondary efficacy endpoints. Continuous outcomes were summarized by the number of observations, mean, standard deviation (SD), median, and range. Categorical outcomes were summarized as count and percentage in each category.

## Results

### Study population

**Figure 1:**
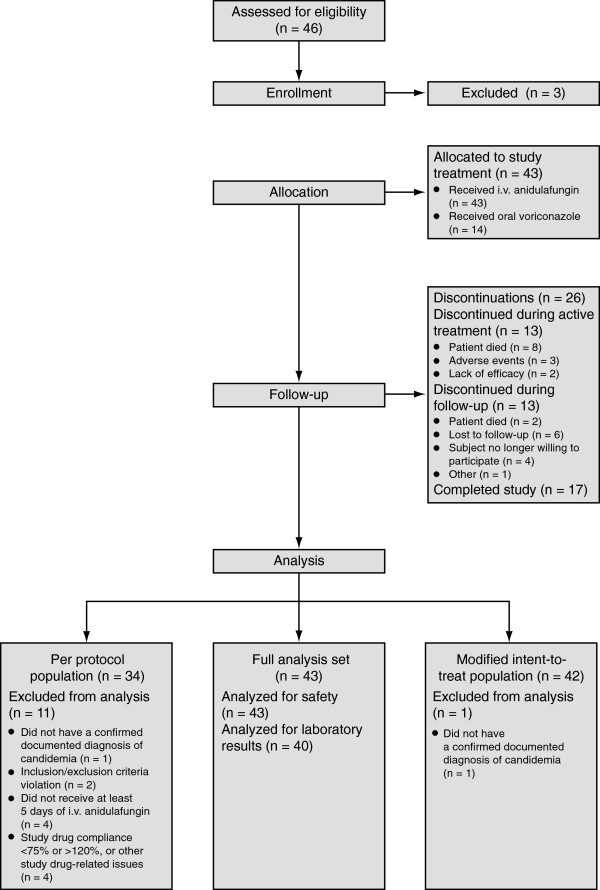
Patient flow chart.

Participants were recruited from 13 study centers across Asia. Due to difficult enrollment, the study was closed before target enrollment could be met. Forty-three patients were studied; however, one patient did not have a confirmed documented diagnosis of candidemia and therefore only 42 patients were included in the MITT population. Eighteen patients were > 65 years, the largest age subgroup. Twenty-one patients had a central venous catheter up to 1 month before baseline (Figure 1, Table 1). At the start of the study, 11 patients had hepatobiliary disorders, 15 had renal and urinary disorders, and 7 patients were receiving chemotherapy.

**Table 1:** Patient baseline characteristics

**Baseline characteristic**	**(n = 43)**
Age, years	
18–44	10
45–64	15
≥ 65	18
Mean ± SD	56.5 (± 18.5)
Range	19–91
Gender, % of patients	
Male	53.5
Female	46.5
Race, n (%)	
Asian	43 (100)
Weight^a^, kg	
Mean ± SD	57.5 (± 12.2)
Range	32.0–84.0
Height^b^, cm	
Mean ± SD	162.4 (± 7.4)
Range	148.0–180.0
Baseline pathogen^c^, n (%)	
*Candida tropicalis*	18 (41.9)
*C. albicans*	14 (32.6)
*C. glabrata*	6 (14.0)
*C. parapsilosis*	4 (9.3)
*C. rugosa*	1 (2.3)
No pathogen isolated	1 (2.3)

^a^Data available from 41 patients.

^b^Data available from 38 patients.

^c^Patients could have more than one pathogen isolated at baseline.

Abbreviation used: *SD*, standard deviation.

The most common *Candida* species isolated was *C. tropicalis* (41.9%, 18/43 patients), followed by *C. albicans* (32.6%, 14/43 patients). Forty-one of the 43 isolates were tested for susceptibility to study treatment. Susceptibility to anidulafungin was 97.6% (40/41) for all *Candida* isolates; one *C. parapsilosis* isolate had an MIC value of > 4 μg/ml. Susceptibility to voriconazole was 90.2% (37/41). For *C. albicans*, two isolates had MIC values > 4 μg/ml, and one isolate had an MIC value between > 1 and 4 μg/ml. For *C. tropicalis*, one isolate had an MIC > 4 μg/ml and another isolate had an MIC value between > 1 and 4 μg/ml.

### Treatment duration

Duration and dose by route of treatment is presented in Table 2. The median duration of i.v. anidulafungin was 10.0 days (range, 1.0 to 37.0 days). Fourteen patients stepped down to oral voriconazole therapy, with an overall median duration of 10 days (range, 4.0 to 38.0 days).

**Table 2:** Duration and dose by route of treatment; ITT population

**Parameter**	**(n = 43)**	
	**Intravenous anidulafungin**	**Oral voriconazole**
Duration, days^a^		
n	43	14
Mean ± SD	11.0 (± 6.81)	12.6 (± 8.89)
Median	10.0	10.0
Range	1.0–37.0	4.0–38.0
Dose, mg/day		
n	43	14
Mean ± SD	117.8 (± 24.09)	364.8 (± 72.86)
Median	110.0	400.0
Range	102.7–200.0	193.8–400.0

^a^Includes only days on which any dose was taken.

Abbreviations used: *ITT*, intent-to-treat; *SD*, standard deviation.

### Efficacy

The global response rate (clinical + microbiological response) at EOT in the MITT population was 73.8% (95% CI, 58.0–86.1 [31/42 patients]) for the primary endpoint, increasing to 79.4% (95% CI, 62.1–91.3 [27/34 patients]) in the per-protocol group (Table 3). Failure occurred in 11.9% of patients (5/42 patients) (Table 3). Secondary endpoints included global response at EOIT, which was 78.6% (33/42 patients) (Table 4).

**Table 3:** Clinical, microbiological, and global responses at the end of all treatment

**Parameter**	**MITT population**	**PP population**
**Clinical response, % [n/N]**		
Success (95% CI)	76.2 (60.5–87.9) [32/42]	82.4 (65.5–93.2) [28/34]
Cure	64.3 [27/42]	67.6 [23/34]
Improvement	11.9 [5/42]	14.7 [5/34]
Failure^a^	4.8 [2/42]	5.9 [2/34]
Indeterminate	14.3 [6/42]	5.9 [2/34]
Not assessed	4.8 [2/42]	5.9 [2/34]
**Microbiological response, % [n/N]**		
Success (95% CI)	81.0 (65.9–91.4) [34/42]	82.4 (65.5–93.2) [28/34]
Eradication	73.8 [31/42]	73.5 [25/34]
Presumed eradication	7.1 [3/42]	8.8 [3/34]
Failure	2.4 [1/42]	2.9 [1/34]
Not assessed	16.7 [7/42]	14.7 [5/34]
**Global response, % [n/N]**		
Success (95% CI)	73.8 (58.0–86.1) [31/42]	79.4 (62.1–91.3) [27/34]
Failure	11.9 [5/42]	8.8 [3/34]
Not assessed	14.3 [6/42]	11.8 [4/34]

Abbreviations used: *CI*, confidence interval; *MITT*, modified intent-to-treat; *PP*, per protocol.

**Table 4:** Clinical, microbiological, and global response rates at various pre-defined efficacy timepoints throughout the study in the MITT population

	**MITT population**		
**Timepoint**	**Clinical response, % [n */ *N] (95% CI)**	**Microbiological response, % [n */ *N] (95% CI)**	**Global response, % [n */ *N] (95% CI)**
End of intravenous treatment	81.0 [34/42]	85.7 [36/42]	78.6 [33/42]
	(65.9–91.4)	(71.5–94.6)	(63.2–89.7)
2 weeks after EOT	83.9 [26/31]	80.6 [25/31]	75.0 [24/32]
	(66.3–94.5)	(62.5–92.5)	(56.6–88.5)
6 weeks after EOT	56.7 [17/30]	56.7 [17/30]	54.8 [17/31]
	(37.4–74.5)	(37.4–74.5)	(36.0–72.7)
12 weeks after baseline	54.8 [17/31]	51.6 [16/31]	50.0 [16/32]
	(36.0–72.7)	(33.1–69.8)	(31.9–68.1)

Abbreviations used: *CI*, confidence interval; *EOT*, end of all treatment; *MITT*, modified intent-to-treat.

Clinical and microbiological responses for the MITT analysis set were also summarized by baseline infection pathogen susceptibility (data not shown). Clinical response rates were slightly lower overall than microbiological response rates. Clinical success was documented in 76.2% of patients, with microbiological success in 81%. Only two patients (4.8%) experienced clinical failure, with one patient (2.4%) having documented pathogen persistence. Clinical assessment was indeterminate for six patients (14.3%) (Table 3). Success rates for the MITT group were 72.2% for *C. tropicalis*, the most common pathogen identified in this study, and 71.4% for *C. albicans* infection. Global success was achieved in 84.6% of patients with previous surgery (11/13 patients), and 58.8% of patients who were over 65 years old. In patients with a central venous catheter, the successful global response was 81.0% (17/21 patients) (Table 5).

**Table 5:** Global response rates in all predefined patients subgroups among MITT patients

**Subgroup**	**Anidulafungin**		
	**Global success %**	**[n */ *N]**	**95% CI**
Neutropenic status^a^			
ANC ≤ 500/mm^3^	50.0	[1/2]	1.3–98.7
ANC > 500/mm^3^	75.7	[28/37]	58.8–88.2
Baseline pathogens^b^			
*Candida albicans*	71.4	[10/14]	41.9–91.6
*C. glabrata*	66.7	[4/6]	22.3–95.7
*C. parapsilosis*	100.0	[4/4]	39.8–100.0
*C. rugosa*	100.0	[1/1]	2.5–100.0
*C. tropicalis*	72.2	[13/18]	46.5–90.3
Previous surgery^c^			
Any surgery	84.6	[11/13]	54.6–98.1
Abdominal surgery	87.5	[7/8]	47.3–99.7
Organ transplantation			
Kidney, liver, or heart	0.0	[0/0]	Not applicable
Elderly status			
Age ≥ 65 years	58.8	[10/17]	32.9–81.6
Renal insufficiency			
Creatinine clearance < 30 ml/min	54.5	[6/11]	23.4–83.3
Use of central venous catheter^c^			
Yes	81.0	[17/21]	58.1–94.6
Receiving chemotherapy^d^			
Yes	71.4	[5/7]	29.0–96.3

^a^ Patients with a missing ANC were not included.

^b^ A patient could have had more than one baseline pathogen.

^c^ Up to 1 month before the baseline visit.

^d^ Up to 3 months before the baseline visit.

Abbreviations used: *ANC*, absolute neutrophil count; *CI*, confidence interval; *MITT*, modified intent-to-treat.

In the MITT population, microbiological success was 80.0% (32/40) in patients with anidulafungin-susceptible isolates and 81.1% (30/37) of those with voriconazole-susceptible isolates; the respective proportions for clinical success were 75.0% (30/40) and 75.7% (28/37). All patients who had isolates resistant to either study agent achieved both clinical and microbiological success. Clinical and microbiological success was reported in all four infections caused by *C. parapsilosis*.

There were no isolates that demonstrated an increase in MICs during the course of the study, including those from patients who were clinical failures where follow-up data were available.

Patients with a successful clinical response had baseline mean β-D-glucan (± SD) levels of 1095.8 ± 968.52 pg/ml, compared to 1447.9 ± 2178.27 pg/ml for patients who suffered clinical failure. At EOT, β-D-glucan levels were 1018.9 ± 783.76 pg/ml in patients who experienced clinical success, and 2917.7 ± 4737.50 pg/ml in patients with clinical failure.

### Safety and tolerability profile

Twelve treatment-related AEs were reported in 10 patients (Table 6). These included diarrhea and rash, both reported in two patients, and the remaining eight treatment-related AEs were each reported in a single patient (chest discomfort, thirst, drug hypersensitivity, recurrence of candidemia, hypokalemia, confusion, insomnia, and choking). All treatment-related AEs were reported as mild-to-moderate in severity.

**Table 6:** Treatment-emergent adverse events

	**Anidulafungin n = 43**	
	**All-causality**	**Treatment-related**
Number of adverse events	150	12
Subjects with adverse events	35	9
Subjects with serious adverse events	16	1
Subjects with severe adverse events	13	0
Subjects discontinued due to adverse events	3	3
Subjects with dose reduced or temporary discontinuations due to adverse events	2	1
Deaths, n (%)		
All causality	10 (23.3)	

The single treatment-related serious AE reported was a recurrence of candidemia (as reported above) in a 54-year-old woman on Day 23, judged by the investigator to be related to voriconazole treatment (after 14 days of voriconazole treatment), which subsequently led to discontinuation of therapy. This patient had *C. glabrata* isolated at baseline, which was susceptible to both voriconazole and anidulafungin (MIC value 0.5 μg/ml for both study drugs). Two other patients also permanently discontinued the study due to AEs (drug hypersensitivity [n = 1] and skin rash [n = 1]), both considered by the investigator to be related to anidulafungin treatment.

There were 10 deaths (23%) during the study, none considered by the investigator to be related to candidemia or to treatment with study drug. In addition, four patients died after completion of treatment, none considered by the investigator to be related to study treatment.

## Discussion

In this study, a regimen of i.v. anidulafungin was assessed for the treatment of documented candidemia in adult Asian patients. The study also included the option to step-down therapy to oral voriconazole. Due to difficult and slow enrollment, the overall sample size was small. However, the efficacy and safety profile of anidulafungin was in line with that previously observed in Western populations.

Efficacy results were generally consistent with those of other studies, including the pivotal trial by Reboli *et al.* that demonstrated better responses with anidulafungin than fluconazole for the treatment of C/IC [12], and an open-label study of anidulafungin dosed at 50 mg, 75 mg, and 100 mg, which reported global success rates (both clinical and microbiological success, i.e. cure/significant improvement of C/IC signs/symptoms and eradication/presumed eradication of *Candida* spp.) of 84%, 90%, and 89%, respectively at EOT, and 72%, 85%, and 83%, respectively, at follow-up [24]. This study suggests that the treatment of candidemia with anidulafungin is as effective in Asian populations as Western populations. The crude mortality rate reported in this study (23%) was also similar to that observed in anidulafungin-treated candidemia patients in North America and Europe [12] and in line with data reported for other echinocandins [8]. These rates are substantially lower than the crude mortality (35% to 60%) associated with candidemia in Asian patients who had not received echinocandins [2,4]. A more recent prospective multicenter Phase IIIb trial (ICE study) demonstrated that anidulafungin was effective and well tolerated with a good safety profile for the treatment of confirmed C/IC in critically ill patients from ≥ 1 of the following subpopulations: post-abdominal surgery, solid tumor, renal insufficiency, hepatic insufficiency, solid organ transplant, neutropenia (neutrophil count < 500 cells/mm^3^), and age ≥ 65 years. The global success rate (both clinical and microbiological success) at EOT (69.5%) was similar to that achieved when anidulafungin is administered in a general population [13,14].

The causative strains isolated in the trial included common *Candida* species found in Asia [2,6]. *C. tropicalis* was the most commonly identified pathogen in this trial. This species is not common in Europe and North America, but data from our study in Asian patients show that it was successfully treated with anidulafungin. Successful responses against *C. albicans*, *C. parapsilosis*, and *C. glabrata* infections were also documented, in line with previous studies [2,6].

As may be expected, the combined rates of microbiological eradication/presumed eradication were slightly higher than the clinical success rates. Global success was achieved in all patients who had baseline isolates resistant to anidulafungin or voriconazole, possibly due to catheter removal eliminating the source of infection. Therefore, the success of treatment of resistant isolates may not be counted as antifungal efficacy. There was no evidence of the development of resistance during active treatment, and there were no isolates that demonstrated an increase in MICs during the course of the study.

Patients with successful clinical responses at EOT had numerically lower mean β-D-glucan levels at baseline than those patients who later experienced clinical failure. This suggests the potential utility of this assay in determining the severity of infection and likely treatment prognosis, and further studies are warranted.

Although investigators had the option to step-down to oral voriconazole therapy after as few as 5 days of treatment with anidulafungin, the median duration of anidulafungin treatment was 11 days and only 14 patients were stepped-down to oral therapy. The duration of i.v. treatment was considerably longer than that reported in another short-course trial (6 days) [25] and was only 3 days shorter than reported in long-course therapy study (14 days) [12]. Possible reasons for this apparent reluctance to step-down to oral therapy at an early timepoint in this study may include the high percentage of non-*albicans* infections, the high proportion of critically ill post-abdominal surgery patients (who may have remained nil-by-mouth for a short period post-operation) and slower clinical response due to an immunocompromised state. It should be noted that during previous clinical studies of anidulafungin for C/IC, patients could be switched to oral fluconazole after completing the i.v. treatment period with anidulafungin [12]. However, due to the continuing increase in fluconazole-resistant strains [1,6] and the high rate of fluconazole resistance in some Asian countries [6], voriconazole only was employed as oral step-down therapy in this study.

AEs were mostly mild or moderate, and consistent with the known favorable safety profile of anidulafungin, with no new safety concerns identified in this regional population [12,26].

The slow enrollment rate in the study could have occurred due to several reasons. The use of fluconazole prophylaxis may have reduced the incidence of C/IC or led to less detection by blood culture, and many candidemic patients with APACHE II score between 20 and 25 were excluded as per the protocol. Other potential shortfalls of this study are the open-label design and the low number of enrolled patients with azole-resistant *Candida* strains (especially *C. krusei*).

Despite the small size, this study retains relevance to clinical practice. First, anidulafungin is easy to use in the clinic as it does not require dose adjustments for renal and hepatic impairment or for concomitant drug use [26-28]. This study included a number of patients who may have required dose adjustment in this study if a different echinocandin had been employed. Secondly, because the study focused on patients with APACHE II scores ≤ 20, the evaluation of clinical efficacy was less likely to be confounded by the severity of the patient’s illness. Finally, anidulafungin demonstrated an efficacy and safety profile in Asian patients that was comparable to clinical trials in Western populations**.**

## Conclusions

Initial data are promising for a course of i.v. anidulafungin for the treatment of C/IC in Asian patients. Although the epidemiology of *Candida* infections was different in Asian patients, the efficacy of anidulafungin was in line with that previously observed in Western populations. Data from the trial showed that treatment of *C. tropicalis* was successful with i.v. anidulafungin. No new safety concerns were identified.

## Abbreviations

AE: Adverse event; APACHE II: Acute Physiology and Chronic Health Evaluation; BID: Twice-daily; CI: Confidence interval; C/IC: Candidemia/invasive candidiasis; EOIT: end of intravenous anidulafungin treatment; EOT: End of all treatment; ITT: Intent-to-treat; i.v.: Intravenous; MIC: Minimum inhibitory concentration; MITT: Modified intent-to-treat; SD: Standard deviation.

## Competing interests

PM has received speaker fees from Pfizer Inc. P-RH has received consultancy fees and honoraria from Pfizer Inc. DT has no relevant conflicts of interest to report. VMC has received honoraria and travel support from Pfizer Inc. VR is a former Pfizer employee and M-LO is a full-time employee of Pfizer Inc.

## Authors’ contributions

VR and MLO conceived and designed the study. PM, PRH, DT, VMC, and VR performed the study. All authors reviewed and approved the final manuscript.

## Pre-publication history

The pre-publication history for this paper can be accessed here:

http://www.biomedcentral.com/1471-2334/13/219/prepub

## Supplementary Material

Additional file 1 List of investigators and corresponding ethics committees or institutional review boards. Click here for file

## References

[B1] Pfaller MA Diekema DJ Epidemiology of invasive candidiasis: a persistent public health problem Clin Microbiol Rev 2007 20 133 163 10.1128/CMR.00029-06 17223626 PMC1797637

[B2] Hsueh PR Graybill JR Playford EG Watcharananan SP Oh MD Ja’alam K Huang S Nangia V Kurup A Padiglione AA Consensus statement on the management of invasive candidiasis in intensive care units in the Asia-Pacific region Int J Antimicrob Agents 2009 34 205 209 10.1016/j.ijantimicag.2009.03.014 19409759

[B3] Pfaller MA Diekema DJ Gibbs DL Newell VA Ellis D Tullio V Rodloff A Fu W Ling TA Results from the ARTEMIS DISK Global Antifungal Surveillance Study, 1997 to 2007: a 10.5-year analysis of susceptibilities of *Candida* species to fluconazole and voriconazole as determined by CLSI standardized disk diffusion J Clin Microbiol 2010 48 1366 1377 10.1128/JCM.02117-09 20164282 PMC2849609

[B4] Chang A Neofytos D Horn D Candidemia in the 21st century Future Microbiol 2008 3 463 472 10.2217/17460913.3.4.463 18651817

[B5] Horn DL Neofytos D Anaissie EJ Fishman JA Steinbach WJ Olyaei AJ Marr KA Pfaller MA Chang CH Webster KM Epidemiology and outcomes of candidemia in 2019 patients: data from the prospective antifungal therapy alliance registry Clin Infect Dis 2009 48 1695 1703 10.1086/599039 19441981

[B6] Pfaller MA Diekema DJ Rinaldi MG Barnes R Hu B Veselov AV Tiraboschi N Nagy E Gibbs DL Results from the ARTEMIS DISK Global Antifungal Surveillance Study: a 6.5-year analysis of susceptibilities of *Candida* and other yeast species to fluconazole and voriconazole by standardized disk diffusion testing J Clin Microbiol 2005 43 5848 5859 10.1128/JCM.43.12.5848-5859.2005 16333066 PMC1317207

[B7] Trick WE Fridkin SK Edwards JR Hajjeh RA Gaynes RP Secular trend of hospital-acquired candidemia among intensive care unit patients in the United States during 1989–1999 Clin Infect Dis 2002 35 627 630 10.1086/342300 12173140

[B8] Horn DL Ostrosky-Zeichner L Morris MI Ullmann AJ Wu C Buell DN Kovanda LL Cornely OA Factors related to survival and treatment success in invasive candidiasis or candidemia: a pooled analysis of two large, prospective, micafungin trials Eur J Clin Microbiol Infect Dis 2010 29 223 229 10.1007/s10096-009-0843-0 20013016

[B9] Han SS Yim JJ Yoo CG Kim YW Han SK Shim YS Lee SM Clinical characteristics and risk factors for nosocomial candidemia in medical intensive care units: experience in a single hospital in Korea for 6.6 years J Korean Med Sci 2010 25 671 676 10.3346/jkms.2010.25.5.671 20436700 PMC2858823

[B10] Ruan SY Hsueh PR Invasive candidiasis: an overview from Taiwan J Formos Med Assoc 2009 108 443 451 10.1016/S0929-6646(09)60091-7 19515624

[B11] Xess I Jain N Hasan F Mandal P Banerjee U Epidemiology of candidemia in a tertiary care centre of north India: 5-year study Infection 2007 35 256 259 10.1007/s15010-007-6144-6 17646917

[B12] Reboli AC Rotstein C Pappas PG Chapman SW Kett DH Kumar D Betts R Wible M Goldstein BP Schranz J Krause DS Walsh TJ Anidulafungin versus fluconazole for invasive candidiasis N Engl J Med 2007 356 2472 2482 10.1056/NEJMoa066906 17568028

[B13] Ruhnke M Paiva JA Meersseman W Pachl J Grigoras I Sganga G Menichetti F Montravers P Auzinger G Dimopoulos G Borges SM Miller PJ Marcek T Kantecki M Anidulafungin for the treatment of candidaemia/invasive candidiasis in selected critically ill patients Clin Microbiol Infect 2012 18 680 687 10.1111/j.1469-0691.2012.03784.x 22404732 PMC3510306

[B14] Dimopoulos G Paiva JA Meersseman W Pachl J Grigoras I Sganga G Montravers P Auzinger G Sa MB Miller PJ Marcek T Kantecki M Ruhnke M Efficacy and safety of anidulafungin in elderly, critically ill patients with invasive Candida infections: a post hoc analysis Int J Antimicrob Agents 2012 40 521 526 10.1016/j.ijantimicag.2012.07.018 22998997

[B15] Blignaut E Pujol C Joly S Soll DR Racial distribution of *Candida dubliniensis* colonization among South Africans J Clin Microbiol 2003 41 1838 1842 10.1128/JCM.41.5.1838-1842.2003 12734214 PMC154709

[B16] Burroughs VJ Maxey RW Levy RA Racial and ethnic differences in response to medicines: towards individualized pharmaceutical treatment J Natl Med Assoc 2002 94 1 26 12401060 PMC2594139

[B17] Matthews HW Racial, ethnic and gender differences in response to medicines Drug Metabol Drug Interact 1995 12 77 91 8591695 10.1515/dmdi.1995.12.2.77

[B18] McCullough MJ Jorge JJ Lejbkowicz F Lefler E Nassar F Clemons KV Stevens DA Genotypic differences of *Candida albicans* and *C. dubliniensis* isolates related to ethnic/racial differences within the same geographic area Mycopathologia 2004 158 39 41 15487318 10.1023/b:myco.0000038432.94844.f7

[B19] Rebora A Guarrera M Racial differences in experimental skin infection with *Candida albicans* Acta Derm Venereol 1988 68 165 168 2454000

[B20] Ostrosky-Zeichner L Alexander BD Kett DH Vazquez J Pappas PG Saeki F Ketchum PA Wingard J Schiff R Tamura H Finkelman MA Rex JH Multicenter clinical evaluation of the (1−>3) beta-D-glucan assay as an aid to diagnosis of fungal infections in humans Clin Infect Dis 2005 41 654 659 10.1086/432470 16080087

[B21] Pfaller MA Diekema DJ Ostrosky-Zeichner L Rex JH Alexander BD Andes D Brown SD Chaturvedi V Ghannoum MA Knapp CC Sheehan DJ Walsh TJ Correlation of MIC with outcome for *Candida* species tested against caspofungin, anidulafungin, and micafungin: analysis and proposal for interpretive MIC breakpoints J Clin Microbiol 2008 46 2620 2629 10.1128/JCM.00566-08 18579718 PMC2519503

[B22] Pfaller MA Diekema DJ Rex JH Espinel-Ingroff A Johnson EM Andes D Chaturvedi V Ghannoum MA Odds FC Rinaldi MG Sheehan DJ Troke P Walsh TJ Warnock DW Correlation of MIC with outcome for *Candida* species tested against voriconazole: analysis and proposal for interpretive breakpoints J Clin Microbiol 2006 44 819 826 10.1128/JCM.44.3.819-826.2006 16517860 PMC1393146

[B23] Clopper CJ Pearson ES The use of confidence or fiducial limits illustrated in the case of the binomial Biometrika 1934 26 404 413 10.1093/biomet/26.4.404

[B24] Krause DS Reinhardt J Vazquez JA Reboli A Goldstein BP Wible M Henkel T Phase 2, randomized, dose-ranging study evaluating the safety and efficacy of anidulafungin in invasive candidiasis and candidemia Antimicrob Agents Chemother 2004 48 2021 2024 10.1128/AAC.48.6.2021-2024.2004 15155194 PMC415613

[B25] Vazquez J Reboli A Pappas P Patterson TF Reinhardt J Chin-Hong P Tobin E Kett D Biswas P Swanson R A phase IV, open-label study evaluating efficacy and safety of intravenous anidulafungin followed by oral azole for the treatment of candidaemia/invasive candidiasis in US/Korean patients [abstract] Clin Microbiol Infect 2011 17 Suppl 4 S33

[B26] Pfizer Inc Eraxis™ (anidulafungin) US physician prescribing information http://labeling.pfizer.com/ShowLabeling.aspx?id=56623676114

[B27] Damle BD Dowell JA Walsky RL Weber GL Stogniew M Inskeep PB In vitro and in vivo studies to characterize the clearance mechanism and potential cytochrome P450 interactions of anidulafungin Antimicrob Agents Chemother 2009 53 1149 1156 10.1128/AAC.01279-08 19029327 PMC2650573

[B28] Dowell JA Stogniew M Krause D Damle B Anidulafungin does not require dosage adjustment in subjects with varying degrees of hepatic or renal impairment J Clin Pharmacol 2007 47 461 470 10.1177/0091270006297227 17389555

