# Supplementary material for: Intravenous anidulafungin followed optionally by oral voriconazole for the treatment of candidemia in Asian patients: results from an open-label Phase III trial
Source: BMC Infect Dis. 2013 May 15;13:219. doi: 10.1186/1471-2334-13-219 (PMC3659089; doi:10.1186/1471-2334-13-219)
Supplement: Additional file 1 — List of investigators and corresponding ethics committees or institutional review boards. [file 1471-2334-13-219-S1.docx]

# A4 LIST OF INVESTIGATORS AND CORRESPONDING ETHICS COMMITTEES OR INSTITUTIONAL REVIEW BOARDS

## India

**Coordinating Investigators:**

<None Entered>

| **Center** | **Principal Investigator** | **Co-Investigator(s)** | **Sub-Investigator(s)** | **Address(es)** | **Institutional Review Board or Ethics Committee Address(es)** |
| --- | --- | --- | --- | --- | --- |
|  |  |  |  |  |  |
| 1005 | Dr. Deepak Talwar |  | Mr. Mir Shad Ali  Dr. Amresh Kumar  Dr. Vipul Mishra | Metro Hospitals & Heart Institutes  X-1, Sector 12  Noida, Uttar Pradesh 201301  INDIA | Metro Ethics Review Board  Metro Hospitals & Heart Institutes  X-1, Sector 12  Noida, Uttar Pradesh 201 301  INDIA |
|  |  |  |  |  |  |
| 1007 | Dr. Girish Chandrakant Rajadhyaksha (Previous PI)  Dr. Rupal Malye |  | Dr. Mohan B. Agarwal  Dr. Mrugraj D. Murtadak  Dr. Nilesh Palasdeokar  Dr. Vaibhav Patil | Lokmanya Tilak Municipal Medical College and Lokmanya Tilak Municipal General Hospital,  Department of Medicine  Babasaheb Ambedkar Road, SION  Mumbai, Maharashtra 400 022  INDIA | Institutional Ethics Commitee  Lokmanya Tilak Municipal Medical College & Lokmanya Tilak Municipal General Hospital  Staff and Research Society, LTM Medical College Building  Room No.17,2nd Floor  Sion  Mumbai, Maharashtra 400 022  INDIA |
|  |  |  |  |  |  |
| 1008 * | Dr. Sriram Sampath |  | Dr. Jayanthi Savio | Department of Intensive Care Unit  St John¿s Medical College Hospital  Sarjapur Road  Bangalore, Karnataka 560034  INDIA | Institutional Ethical Review Board  St. John's Medical College and Hospital  Sarjapur Road  Bangalore, Karnataka 560 034  INDIA |
|  |  |  |  |  |  |
| 1009 * | Dr. Simran Singh |  | Dr. Umakant R. Bhutada  Dr. Farhad N. Kapadia | P.D. Hinduja National Hospital & Medical Research Centre  Veer Savarkar Marg  Mahim  Mumbai, Maharashtra 400 016  INDIA | National Health & Education Society Clinical Research & Ethics Committee  P.D. Hinduja National Hospital & Medical Research Centre  Veer Savarkar Marg, Mahim  Mumbai, Maharashtra 400 016  INDIA |
|  |  |  |  |  |  |
| 1010 | Dr. Dinesh Jain |  | Mr. Vijay Kumar Gupta  Ms. Anju Jain | Dayanand Medical College and Hospital, Department of Medicine  Tagore Nagar, Civil Lines  Ludhiana, Punjab 141 001  INDIA | Drug Trial Ethical Committee, Dayanand Medical College & Hospital  Tagore Nagar, Civil Lines,  Ludhiana, Punjab 141 001  INDIA |
|  |  |  |  |  |  |
| 1026 | Dr. Parthiv M. Mehta |  | Dr. Roocha Majmundar Mehta  Dr. Jigar J. Parikh | Mehta Hospital & Cardiopulmonary Care Center  2nd floor, Siddhachal Complex  Nr. Doordarshan Kendra  Drive-in-Road  Ahmedabad, Gujarat 380 054  INDIA | Safe Search Independent Ethics Committee  Siddhachal Complex, Nr. Doordarshan Kendra  Thaltej  Ahmedabad, Gujarat 380 054  INDIA |
|  |  |  |  |  |  |

## Philippines

**Coordinating Investigators:**

Dr. Racquel Victoria Ecarma

| **Center** | **Principal Investigator** | **Co-Investigator(s)** | **Sub-Investigator(s)** | **Address(es)** | **Institutional Review Board or Ethics Committee Address(es)** |
| --- | --- | --- | --- | --- | --- |
|  |  |  |  |  |  |
| 1002 | Dr. Vilma M. Co |  | Jenny Lynd Calapati  Dr. Carmenchu Marie Echiverri | Makati Medical Center  Room 378  2 Amorsolo St.  Legaspi Village, Makati City, 1200  PHILIPPINES | Makati Medical Center  Institutional Review Board  2 Amorsolo St.  Legaspi Village, Makati City, 1200  PHILIPPINES |
|  |  |  |  |  |  |
| 1003 * | Dr. Danilo Castro |  | Karen Joe Lacida | East Avenue Medical Center  Department of Medicine  East Ave.,  Quezon City, 1100  PHILIPPINES | East Avenue Medical Center  Institutional Ethics Review Board  East Avenue, Diliman  Quezon City, 1101  PHILIPPINES |
|  |  |  |  |  |  |
| 1004 * | Dr. Remedios Coronel |  | Dr. Rhona Bergantin  Anna Liza Cuaresma | Room 6011  Section of Infectious and Tropical Diseases  University of Sto. Tomas Hospital  Espana St.  Manila, 1000  PHILIPPINES | University of Sto. Tomas Hospital - Institutional Review Board  Room 432, Clinical Division Building  A.H. Lacson Street, España  Manila,  PHILIPPINES |
|  |  |  |  |  |  |
| 1012 * | Dr. Cecilia Montalban |  | Arthur Dessi Estrada Roman | Manila Doctors Hospital  Room 412  667 United Nations Ave.  Ermita, Manila 1000  PHILIPPINES | Technical and Ethical Review Board  Manila Doctors Hospital  667  United Nations Avenue  Ermita  Manila City,  PHILIPPINES |
|  |  |  |  |  |  |
| 1013 * | Dr. Adrian C. Pena (Previous PI)  Dr. Remedios Coronel |  | Minette Rosario | St. Luke's Medical Center  Infection Control Office  279 E. Rodriguez Sr. Boulevard  Quezon City, Philippines  PHILIPPINES | Institutional Ethics Review Board  Research and Biotechnology Division St. Luke's Medical Center  279 E. Rodriguez Sr. Blvd.,  Quezon City, 1102  PHILIPPINES |
|  |  |  |  |  |  |
| 1014 * | Dr. Adrian C. Pena |  | Melquiedes Marino Pua | Philippine Heart Center  Department of Pathology  East Avenue, Diliman  Quezon City, 1101  PHILIPPINES | Philippine Heart Center  Institutional Review Board  Medical Arts Building  East Avenue  Quezon City, 1101  PHILIPPINES |
|  |  |  |  |  |  |
| 1019 * | Dr. Cecilia Montalban |  | Dr. Maria Luna Parreno | Philippine General Hospital  Infectious Diseases Section  2nd Floor, ER complex UP-PGH Medical Center  Taft Avenue  Manila, 1000  PHILIPPINES | Research Implementation and Development Office  UP College of Medicine  547 Pedro Gil st  Ermita, Manilla 1000  PHILIPPINES |
|  |  |  |  |  |  |
| 1027 * | Dr. Myrna T. Mendoza |  |  | National Kidney and Transplant Institute  Department of Internal Medicine  Rm. 3237  East Avenue  Quezon City, 1101  PHILIPPINES | Research Ethics Committee  National Kidney Institute  East Avenue  Quezon City,  PHILIPPINES |
|  |  |  |  |  |  |

## Taiwan

**Coordinating Investigators:**

<None Entered>

| **Center** | **Principal Investigator** | **Co-Investigator(s)** | **Sub-Investigator(s)** | **Address(es)** | **Institutional Review Board or Ethics Committee Address(es)** |
| --- | --- | --- | --- | --- | --- |
|  |  |  |  |  |  |
| 1001 | Dr. Pan-chyr Yang |  | Wei-Ting Chen  Yee-chun Chen  Dr. Po-ren Hsueh  Chi-Ying Lin  Chia-Ying Liu MD | Department of Inernal Medicine  No. 7, Chung-Shan South Road  Taipei, 100  TAIWAN | National Taiwan University Hospital, Ethics Committee  7 Chung Shan South Road  Taipei, 10012  TAIWAN |
|  |  |  |  |  |  |
| 1011 | Dr. Jen-hsien Wang |  | Chih-Yu Chi | China Medical University Hospital  Dept of Internal Medicine, Section of Infectious Diseases/  No. 2 Yuh Der Road  Taichung, 404  TAIWAN | China Medical University Hospital, The Institutional Review Board  2 Yu Der Road  Taichung, Taiwan 404  TAIWAN |
|  |  |  |  |  |  |
| 1016 | Wen-Chien Ko |  | Chi-Jung Wu | Department of Infectious Disease,National Cheng Kung University Hospital  No. 138, Cheng-Li Road  Tainan, 704  TAIWAN | Human Experiment and Ethics Committee National Cheng Kung University Hospital  138 Sheng Li Road  Tainan, 704  TAIWAN |
|  |  |  |  |  |  |
| 1025 | Dr. Chun-Hsing Liao |  |  | Far-Eastern Memorial Hospital/ Infectious Disease Section  No. 21, Nan-Ya S. Rd., Sec.2  Pan-Chiao, Taipei 220  TAIWAN | Far Eastern Memorial Hospital, Research Ethics Review Committee  21, Nan-Ya S. Rd., Sec.2 Pan-Chiao  Taipei,  TAIWAN |
|  |  |  |  |  |  |

## Thailand

**Coordinating Investigators:**

<None Entered>

| **Center** | **Principal Investigator** | **Co-Investigator(s)** | **Sub-Investigator(s)** | **Address(es)** | **Institutional Review Board or Ethics Committee Address(es)** |
| --- | --- | --- | --- | --- | --- |
|  |  |  |  |  |  |
| 1017 | Prof. Khuanchai Supparatpinyo |  | Dr. Nontakan Nuntachit  Dr. Parichat Salee | Division of Infectious Diseases and Tropical Medicine, Department of Medicine, Faculty of Medicine,  Maharaj Nakorn Chiang Mai Hospital, Chiang Mai University  110 Intavaroros Street  Amphoe Mueang, Chiang Mai 50200  THAILAND | Research Ethics Committee, Chiang Mai University  Research Ethics Committee, Faculty of Medicine, Chiang Mai University  110  Intavaroros Street  Amphoe Mueang, Chiang Mai 50200  THAILAND |
|  |  |  |  |  |  |
| 1018 | Assoc. Prof. Piroon Mootsikapun |  | Assoc. Prof. Siriluck Anunnatsiri  Prof. Ploenchan Chetchotisakd | Division of Infectious Diseases and Tropical Medicine, Department of Medicine  Faculty of Medicine, Srinagarind Hospital, Khon Kaen University  123 Mitraparb Road  Amphoe Mueang, Khon Kaen 40002  THAILAND | The Khon Kaen University Ethics Committee for Human Research  Faculty of Medicine, Khon Kaen University  Dean Office 6 floor  123 Mitraphap Road  Khon Kaen, 40002  THAILAND |
|  |  |  |  |  |  |
| 1020 * | Assoc.Prof. Winai Ratanasuwan |  | Dr. Thanomsak Anekthananon  Dr. Wichai Techasathit | Department of Preventive and Social Medicine,  Faculty of Medicine Siriraj Hospital, Mahidol University  2 Prannok Road  Bangkoknoi, Bangkok 10700  THAILAND | Siriraj Ethics Committee  2 Bangkoknoi, Siriraj Hospital, Mahidol University  Prannok Road  Bangkok, 10700  THAILAND |
|  |  |  |  |  |  |
| 1021 | Dr. Chusana Suankratay |  | Dr. Chalinee Laosakul  Dr. Opass Putcharoen  Dr. Gompol Suwanpimolkul | Division of Infectious Diseases, Department of Medicine  Faculty of Medicine, Chulalongkorn University,  1873 Rama IV Road  Pathumwan, Bangkok 10330  THAILAND | The Institutional Review Board of Faculty of Medicine, Chulalongkorn University, Bangkok  1873 Rama 4 Road, Pathumwan  Bangkok, 10330  THAILAND |
|  |  |  |  |  |  |
